# The Predictive Role of Inflammatory Biomarkers and Their Correlation with the Biochemical Profile in Patients with Vasculopathy Undergoing Surgery

**DOI:** 10.3390/ijms252211989

**Published:** 2024-11-08

**Authors:** Orsolya-Zsuzsa Akácsos-Szász, Sándor Pál, Kinga-Ilona Nyulas, Mónika Szilveszter, Zsuzsánna Simon-Szabó, Lóránd Dénes, Erzsébet Májai, Adina Huțanu, Adina Stoian, Mariana Cornelia Tilinca, Enikő Nemes-Nagy

**Affiliations:** 1 Doctoral School, Faculty of Medicine, George Emil Palade University of Medicine, Pharmacy, Science, and Technology of Târgu Mureş, 540139 Targu Mures, Romania; 2 Department of Laboratory Medicine, Department of Transfusion Medicine, Medical School, University of Pécs, 7622 Pécs, Hungary; 3 Clinic of Plastic Surgery, Mureș County Emergency Hospital, 540136 Targu Mures, Romania; 4 Department of Pathophysiology, Faculty of Medicine, George Emil Palade University of Medicine, Pharmacy, Science, and Technology of Târgu Mureş, 540139 Targu Mures, Romania; adina.stoian@umfst.ro; 5 Department of Anatomy, Embryology, Faculty of Medicine, George Emil Palade University of Medicine, Pharmacy, Science, and Technology of Târgu Mureş, 540139 Targu Mures, Romania; lorand.denes@umfst.ro; 6 Department of Toxicology and Biopharmacy, Faculty of Pharmacy, George Emil Palade University of Medicine, Pharmacy, Science, and Technology of Târgu Mureş, 540139 Targu Mures, Romania; 7 Center for Advanced Medical and Pharmaceutical Research, Department of Laboratory Medicine, Faculty of Medicine, George Emil Palade University of Medicine, Pharmacy, Science, and Technology of Târgu Mureş, 540139 Targu Mures, Romania; 8 Department of Internal Medicine I, Faculty of Medicine in English, George Emil Palade University of Medicine, Pharmacy, Science, and Technology of Târgu Mureş, 540139 Targu Mures, Romania; 9 Department of Chemistry and Medical Biochemistry, Faculty of Medicine in English, George Emil Palade University of Medicine, Pharmacy, Science, and Technology of Târgu Mureş, 540139 Targu Mures, Romania; eniko.nemes-nagy@umfst.ro

**Keywords:** amputation, biochemical parameters, cytokines, diabetes mellitus, IL-6, inflammation, malondialdehyde, perioperative, TNF-α, vasculopathy

## Abstract

Inflammation is involved in the pathomechanism of vascular diseases. Pro-inflammatory cytokines are important in perioperative monitoring. The aim of the study was the perioperative assessment of biochemical tests and inflammatory markers in patients with vasculopathy, focusing on the identification of subjects prone to complications. The study was performed between 2020 and 2023 at the Clinical County Hospital in Târgu Mureș on enrolled diabetic and non-diabetic patients with vasculopathy and lower limb surgery (amputation or necrectomy). Pre- and postoperative inflammatory markers, biochemical, and hematological tests (n = 62) were performed. Positive correlation was found between preoperative C-reactive protein (CRP) and interleukin-6 (IL-6) levels, and between preoperative triglyceridemia and glycemia/cholesterolemia. Positive correlation was present between pre- and postoperative values of IL-6, tumor necrosis factor-alfa (TNF-α), CRP, and fibrinogen. Preoperative TNF-α values positively correlated with malondialdehyde (MDA) levels, postoperative TNF-α values with transaminase enzymes. Diabetic patients presented higher IL-6 results compared to non-diabetic subjects. We can conclude that dynamic assessment of inflammatory markers is appropriate for monitoring perioperative course. Half of the subjects presented moderately increased preoperative IL-6 levels, and one quarter had critically high values, which might predict prolonged hospitalization. The assessment of oxidative stress, inflammatory markers and biochemical parameters enables the identification of patients prone to complications, so they can benefit from more complex management.

## 1. Introduction

Globally, cardiovascular diseases are the leading cause of death. Research suggests that inflammation is a crucial factor in the pathophysiology of vascular diseases. Atherosclerosis, a chronic inflammatory condition affecting the arterial wall in large and medium-sized vessels, is the primary underlying cause of many cardiovascular diseases, including coronary artery disease, stroke, and peripheral artery disease [1,2,3].

Disruption of endothelial barrier function can result in vascular hyperpermeability and edema in a variety of conditions, such as sepsis, ischemia, and trauma. Endothelial permeability can be altered by a range of physiological and pathophysiological stimuli, including acute and chronic changes induced by inflammatory mediators such as TNF-α and vascular endothelial growth factor (VEGF) on endothelial cells [4].

Atherosclerosis is a chronic inflammatory disease of the vessels that is primarily triggered by the interplay between endothelial cells and immune cells. In recent decades, there has been significant interest in the potential role of inflammatory mediators as common risk factors in atherosclerosis. These mediators can influence the levels of low-density lipoproteins (LDLs) and triglyceride-rich lipoproteins, and they alter the structure of the arterial wall, which leads to the attraction of leukocytes. The concept of inflammation as a promoter of atherosclerosis provides additional mechanisms by which traditional risk factors, such as LDL, can induce the development of atherosclerotic disease and its complications [5].

Early-stage atherosclerosis is characterized by endothelial injury, abnormal lipid metabolism, and hemodynamic damage. Flow-mediated inflammatory changes activate endothelial cells, which express inflammatory factors, attracting lymphocytes and monocytes. Inflammation is the primary response to injury, with cytokines such as TNF-α and IL-6 playing a significant role. TNF-α is the most critical mediator, as it has prolonged action with inducing the inflammatory response and is responsible for the synthesis of endothelial glycoproteins. The build-up of a significant amount of low-density lipoproteins (LDL) that are converted to oxidized LDL (ox-LDL) in the vascular intima contributes to the development of atherosclerotic plaques. IL-6 is a pleiotropic cytokine that regulates both acute-phase and chronic inflammation and plays a critical role in the innate and adaptive immune system. It may serve as a potential marker for predicting the vulnerability of atherosclerotic plaques. CRP is mainly produced by the liver when stimulated by IL-6, IL-1β, and TNF-α, and can provide valuable information in diagnosing and monitoring patients with atherosclerosis [6,7].

Genetic polymorphisms in the TNF-α and IL-10 genes play a crucial role in the development of insulin resistance, type 2 diabetes mellitus (T2DM), and its complications. TNF-α serves as a regulator of inflammation, apoptosis, cytotoxicity, and the production of cytokines such as IL-1 and IL-6. The development of insulin resistance is closely linked to dyslipidemia and elevated levels of pro-inflammatory cytokines such as TNF-α and IL-6 [3,8].

A decrease in IL-6 levels was observed in type 2 diabetic patients treated with a glucagon-like peptide 1 (GLP-1) receptor agonist, which correlated with a reported lower waist circumference, thus presenting a beneficial effect in reducing cardiovascular risk [9].

Peripheral diabetic neuropathy (PDN) is a common complication of diabetes leading to sensory and motor deficits. PDN can lead to a loss of protective sensation, which in turn increases the risk of developing foot ulcers, especially in patients with poor glycemic control and/or peripheral arterial disease (PAD) [10,11].

Both IL-6 and TNF-α have been implicated in the pathogenesis of PDN. Elevated levels of these cytokines have been found in the peripheral nerves and skin of diabetic patients with PDN [12]. However, elevated IL-6 levels were associated with PDN in younger diabetic patients only, while in the case of elderly patients, the measured IL-6 concentrations were less elevated [13]. IL-6 and CRP could also be used as early diagnostic biomarkers for obesity-related peripheral polyneuropathy in non-diabetic patients [14].

In addition to their role in PDN, IL-6 and TNF-α may also contribute to the pathogenesis of diabetic foot ulcers. Elevated levels of these cytokines have been detected in the serum and wound discharge of diabetic patients with foot ulcers, and it is thought that they contribute to the development and progression of ulcers by promoting inflammation, impairing angiogenesis, and delaying the healing process [15,16]. Furthermore, TNF-α can induce the production of reactive oxygen species (ROS), leading to endothelial dysfunction and impaired blood flow to the affected tissues, which can additionally impair the healing of ulcers. TNF-α can also stimulate the production of other cytokines, including IL-6, which can amplify the inflammatory response [17,18].

PDN, the most common diabetic microvascular complication, has varying incidence and prevalence rates in different epidemiological studies. Subjects with high levels of TNF-α, IL-6, and intercellular adhesion molecules (ICAM-1) had a significantly higher incidence of PDN compared to the patients with low levels of these cytokines. The role of IL-6 in PDN is not yet fully understood, but TNF-α can stimulate mononuclear cells to produce cytokines, including IL-6, and these two cytokines concomitantly contribute to the development of PDN. Elevated levels of pro-inflammatory cytokines could serve as a useful predictor of PDN incidence [19].

Higher levels of IL-6 and CRP were both linked to an increased risk of soft tissue infections and may serve as predictors of lower-limb amputation. Furthermore, elevated levels of these cytokines may indicate a greater risk of postoperative mortality [20]. Moreover, in a study of diabetic patients, Sun et al. found that increased CRP, IL-6, and TNF-α concentrations were associated with moderate-to-severe soft tissue infections and their levels decreased markedly after antibiotic therapy [21]. Another study of 37 midfoot amputations and bypass surgical procedures led by Sapienza et al. monitored the inflammatory markers of the patients, including IL-6 and TNF-α. The increased levels of these cytokines had predictive values of not healing wounds, necessitating major amputation [22]. Gremmels et al. studied patients suffering from severe limb ischemia, investigating a panel of inflammatory cytokines to identify biomarkers for major events in these patients. IL-6 was significantly higher in patients with major amputation or death [23]. Given the limited number of studies conducted on IL-6 and TNF-α in patients undergoing amputation, their exact role during these procedures is not yet fully understood. However, the available data suggest that IL-6, CRP, and TNF-α measurements in diabetic foot ulcers could be valuable tools for patient risk stratification, as well as predictors for lower-limb amputation and perioperative risk of morbidity and mortality.

The aim of the study was the perioperative assessment of pro-inflammatory cytokines, and other inflammatory and biochemical parameters, to identify correlations between these values, identify possible predictive factors, and to investigate the differences between the diabetic and non-diabetic subgroups. Data on immune activation, low-grade chronic systemic inflammation, and lower limb vasculopathy are not well reported in the literature, most of them relating to the diabetic foot; therefore, we considered that a study involving both diabetic and non-diabetic patients would be of value. The secondary aim of this study was to identify patients with intense inflammation and high levels of oxidative stress, as these subjects are predisposed to complications, prolonged hospitalization, and need complex management.

## 2. Results

The mean age of the subjects was 71.13 ± 12.86 (SD) years, 71% of them were male. Out of the subjects, 68% also had type 2 diabetes mellitus and 90% were diagnosed with hypertension. Of the total cases, 77% had dyslipidemia and 22% were smokers.

A positive correlation was found between preoperative CRP and IL-6 levels (r = 0.4419, *p* = 0.0238). No correlation could be observed between preoperative concentrations of TNF-α and IL-6 (r = −0.2613, *p* = 0.1631), TNF-α and CRP (r = −0.2310, *p* = 0.2563), CRP and fibrinogen (r = 0.1187, *p* = 0.5635), ESR and IL-6 (r = 0.1771, *p* = 0.3867), fibrinogen and TNF-α (r = −0.0359, *p* = 0.8562), ESR and TNF-α (r = −0.2396, *p* = 0.2385), leukocyte number and ESR (r = −0.0029, *p* = 0.9887), or leukocyte number and fibrinogen levels (r = 0.0638, *p* = 0.7425). Postoperative concentrations of TNF-α and IL-6 did not show significant difference either (r = −0.1492, *p* = 0.4314).

There was a positive correlation between preoperative and postoperative IL-6 levels (*p* < 0.0001, r = 0.8121), TNF-α levels (*p* < 0.0001, r = 0.8261), preoperative and postoperative serum CRP concentrations (r = 0.5842, *p* = 0.0022), and fibrinogen levels (r = 0.4317, *p* = 0.0352). The average preoperative IL-6 level in the diabetic subgroup was 33.05 ± 40.52 (SD) pg/mL, the mean value of this parameter in the non-diabetic subgroup was considerably less, 14.48 ± 6.61 (SD) pg/mL, and the difference is not significant (*p* = 0.0920). The results for the postoperative levels of this cytokine showed the following statistical difference: the mean level of IL-6 in diabetic patients was 24.82 ± 28.06 (SD) pg/mL, which was significantly greater (*p* = 0.0460) than the average value recorded in the non-diabetic subgroup (13.19 ± 7.89 (SD) pg/mL).

A smaller, non-significant difference (*p* = 0.5446) was seen between preoperative TNF-α levels in the diabetic (21.08 ± 8.52 (SD) pg/mL) versus non-diabetic (24.33 ± 13.63 (SD) pg/mL) subgroup. When comparing the postoperative mean values of this parameter, we observed similar findings between the two subgroups: the mean TNF-α value was 22.72 ± 12.41 (SD) pg/mL in diabetic and 19.45 ± 11.97 (SD) pg/mL in non-diabetic subjects (*p* = 0.2540). There were similar results between pre- and postoperative TNF-α levels (22.24 ± 10.41 pg/mL vs. 22.10 ± 12.21 pg/mL, *p* = 0.913) across the overall cohort.

Preoperative CRP mean values were double in diabetic subjects (8.74 ± 7.82 (SD) mg/dL) compared to the non-diabetic subgroup (4.82 ± 4.61 (SD) mg/dL), but the difference was not significant (*p* = 0.2267). The difference between these subgroups was found to be minor for the postoperative levels of the inflammatory marker: the mean CRP concentration was 11.57 ± 20.28 (SD) mg/dL in the diabetic subgroup, compared to 10.39 ± 5.06 (SD) mg/dL in the non-diabetic subgroup (*p* = 0.8189).

No significant difference could be observed between the preoperative (10.64 ± 3.80 × 10^9^/L) and postoperative (9.88 ± 3.18 × 10^9^/L) values of the leukocyte number (*p* = 0.218). No significant modification was seen (*p* = 0.7728) between the preoperative (84.19 ± 43.41 mm/h) and postoperative ESR results (75.68 ± 38.49 mm/h).

Regarding the measured biochemical parameters, positive correlation was present between preoperative levels of glycemia and triglyceridemia (r = 0.5720, *p* = 0.0012) and between preoperative levels of cholesterolemia and triglyceridemia (r = 0.5497, *p* = 0.0020). No correlation could be found between preoperative serum glucose levels and the measured inflammatory markers.

The average values of the pre- and postoperative biochemical parameters are presented in Table 1. No significant differences could be observed between the pre- and postoperative values.

There was no significant difference (*p* = 0.6220) between preoperative and postoperative IL-6 levels (preoperative concentrations 23.43 ± 29.36 (SD) pg/mL, postoperative concentrations 21.84 ± 24.54 (SD) pg/mL).

The postoperative liver function test results showed positive correlation with the postoperative TNF-α values, but there was no correlation between pre-and postoperative IL-6 values and the measured biochemical parameters. The parameters, the correlation coefficients, and the *p* values are presented in Table 2.

The positive correlation between age and postoperative IL-6 levels (r = 0.488, *p* = 0.006) was significant in both subgroups.

Of the subjects, 27% presented preoperative IL-6 values exceeding 6 pg/mL and 13% had preoperative TNF-α values higher than 10 pg/mL. Regarding postoperative test results, 17% of the subjects presented a serum IL-6 level above 6 pg/mL and in 10% of the subjects, TNF-α values exceeded 10 pg/mL. In the case of serum TNF-α concentration, the highest pre- and postoperative levels were 51.52 pg/mL and 61.71 pg/mL, respectively.

Across the overall cohort, the highest preoperative IL-6 value was 139.26 pg/mL, while the highest postoperative value was 132.21 pg/mL. Of the subjects, 23% had preoperative IL-6 concentrations exceeding 35 pg/mL, while the postoperative results showed only 7% of the IL-6 levels were over this threshold.

**Figure 1:**
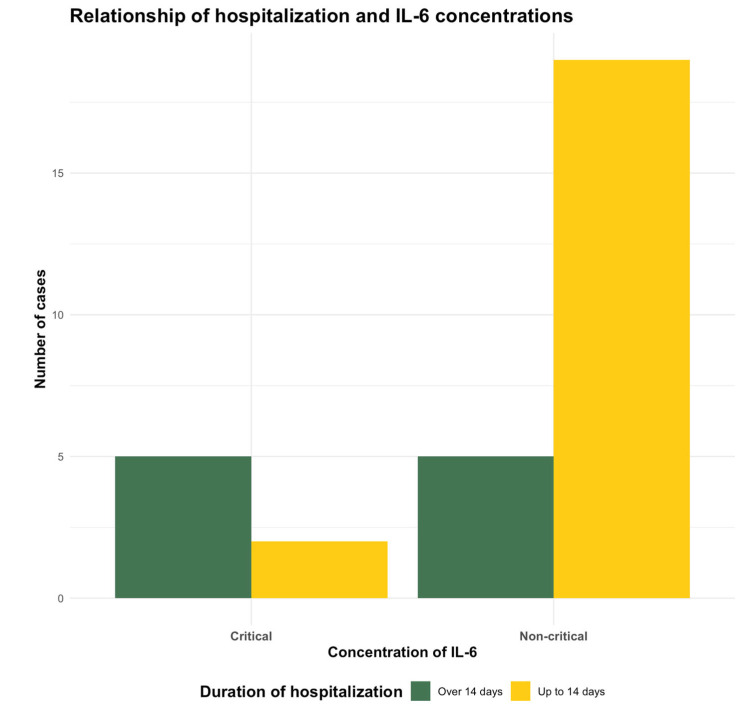
Distribution of hospitalization length depending on preoperative IL-6 values.

A significant difference was found in the distribution of cases with prolonged hospitalization depending on the preoperative IL-6 serum concentrations: those with critical values of IL-6 (exceeding 35 pg/mL) were prone to staying in the hospital for more than 2 weeks (*p* = 0.0394) (Figure 1).

Serum malondialdehyde concentration was higher after the surgical intervention (133.68 ± 166.96 ng/mL) compared to the initial values (114.48 ± 50.98 ng/mL), but the difference was not statistically significant (*p* = 0.5807). A positive correlation was found between preoperative MDA and TNF-α values (r = 0.4670, *p* = 0.0214).

The distribution of shorter (up to 2 weeks) or longer admission periods (>2 weeks) between patients with normal or higher neurophil to lymphocyte ratio (NLR) was as follows: any patient who has stayed more than 2 weeks (n = 10) had NLR under 3. The 14 patients with shorter admission had NLR under 3 and 7 patients were had NLR over 3 (*p* = 0.0661). Comparing the mean value of NLR of patients with longer hospitalization (1.53 ± 0.57) and shorter hospitalization (3.88 ± 4.94) at the surgical unit, significant difference was seen (*p* = 0.0432).

There was no significant difference in the length of hospital admission between diabetic and non-diabetic patients with vasculopathy.

## 3. Discussion

Previously published data suggested that diabetic patients, especially if obese, tend to have higher IL-6 and TNF-α levels. TNF-α levels are directly related to the weight and body mass index (BMI) of patients with diabetes and are greater in obese people [1]. It is hypothesized that in this group of patients, major surgeries may carry higher postoperative risks, due to the increased inflammatory state. The present study partially supports this hypothesis, as the postoperative IL-6 concentrations of the studied group were significantly higher in diabetic patients compared to the non-diabetic subjects. Chronic inflammation and impaired wound healing are hallmarks of diabetic ulcers, and these processes involve the dysregulation of multiple cytokines, including IL-6 and TNF-α. Several studies have reported that elevated IL-6 levels in patients with lower-limb ischemia requiring revascularization surgery may indicate higher postoperative morbidity and mortality, as well as an increased risk of amputation. This is of particular concern in diabetic patients, as they are already at higher risk of developing postoperative complications. The continuation of our research would be crucial to provide more definitive data on the potential risks associated with major surgeries in diabetic and obese patients. The present study is important in highlighting the potential risks of major surgery in diabetic and/or obese patients, particularly in relation to their increased inflammatory state. This information can be used to improve patient selection for surgery and optimize perioperative management strategies. It also underscores the importance of routine monitoring of inflammatory markers in these patients to identify those at higher risk for postoperative complications. This study adds to the growing body of evidence suggesting that elevated IL-6 levels may be a risk factor for poor outcomes following major surgeries in diabetic and/or obese patients. However, due to the limited number of subjects in our study, further research is needed to confirm these findings and to investigate potential strategies for mitigating these risks [20,21,22,23].

In diabetes mellitus, the increase in glucose levels is correlated with the increased recruitment of macrophages followed by the intensification of the secretion of TNF-α and cytokines [24]. The secretion of IL-6 and TNF-α influences the production of CRP in the liver [25]. Our study could not identify a statistically significant difference between the preoperative CPR levels of diabetics and non-diabetic subjects, although the CRP values obtained were twice as high as in the diabetic subgroup compared to the non-diabetic one.

Inflammation is also important for the initiation and progression of peripheral arterial disease (PAD) and the inflammatory mediators are similar to those involved in the development of other atherosclerotic diseases, such as coronary heart disease [24]. Low-grade inflammation is involved in all phases of PAD, starting with the development of atherosclerotic plaques and until the appearance of its complications with rupture and thrombosis [25]. Inflammation and diabetes are part of the metabolic syndrome (MS), which is characterized by the coexistence of obesity, hypertension, dyslipidemia, and hyperglycemia in an individual, increasing the risk of developing cardiovascular disease (CVD) [6]. Elevated levels of pro-inflammatory factors, including TNF-α, IL-6, and ICAM-1, can have a predictive role for the development of diabetic neuropathy in type 2 diabetic patients [19]. Smoking, single nucleotide polymorphisms (SNPs) of the genes encoding pro-inflammatory molecules, infections, obesity, hypertension, and autoimmune diseases such as rheumatoid arthritis and systemic vascular lupus involve a systemic inflammatory response and are correlated with the occurrence of PAD [24,26].

In a prospective case–control study that included apparently healthy men, published in 1998, Ridker et al. found that the relative risk of developing PAD increases significantly with increasing CRP, so that men with CRP around 2.1 mg /L had a double risk compared to men with CRP around 0.55 mg/L and this result was independent of other risk factors such as DM, arterial hypertension, BMI, and family history of atherosclerotic diseases [27]. Another study conducted 3 years later, which included 221 young women diagnosed with PAD and 475 healthy control women, demonstrated that the increased level of CRP and the existence of certain types of symptomatic infections were associated with the presence of PAD [28].

Levels of markers like Il-6, IL-8, TNF-α, pentraxin-3, and calprotectin were reported to be increased in PAD patients compared to the control group [27]. In addition, IL-6 and TNF-α were also associated with the severity of PAD evaluated by clinical scales and by the ankle-brachial index (ABI) [29,30,31]. Overall, the pathomechanism of the development and progression of diabetic lower limb ulcers involves the role that IL-6 and TNF-α play in stimulating inflammation, oxidative stress, and impaired wound healing. In diabetic patients with peripheral neuropathy, these cytokines can further increase the risk of developing foot ulcers due to the loss of protective sensation and impaired neurovascular function. Il-6 has been shown to be a predictor of functional outcomes and disease progression. In the Edinburgh Artery Study, the IL-6 level correlated with disease progression and ABI changes at 5 years and at 12 years of follow-up, and CRP levels correlated with progression and ABP changes at 12 years [32].

Pro-inflammatory cytokines, such as CRP, TNF-α, and IL-6, are increased after surgery, which leads to increased vascular permeability, which could be an important mechanism in the postoperative decrease in serum albumin levels in these patients [7]. These cytokines did not show significant changes after the surgery in our study group.

The results of this study showed that significant short-term modifications of IL-6 and TNF-α concentrations did not occur in our cohort.

The effect of IL-6 and TNF-α on postoperative morbidity and mortality is still not fully known. Some of the relatively few available publications regarding these cytokines suggest that IL-6 and TNF-α may represent a risk for the patients, while other sources associate anti-TNF-α therapy with worse postoperative outcomes in the case of patients receiving such therapies [33]. Brocca and colleagues conducted a study involving 122 patients following cardiac surgery where IL-6 and procalcitonin levels were measured on the second postoperative day and reported that IL-6 was highly predictive of overall mortality 30 days after surgery (*p* < 0.05) [34]. Guo, S. et al. found that secondary to angioplasty and stent placement, an inflammatory response is induced, and IL-6 is a powerful predictor of restenosis at 6 months [35]. Further studies are needed for the assessment of these cytokines’ effect on postoperative morbidity and mortality.

The diabetic foot appears because of damage to peripheral nerves (neuropathy) and blood vessels (atherosclerosis) and changes in the bone anatomy of the foot. Some of the major complications include the development of foot ulcers with consequential amputations [36,37]. It is thought that IL-6 and TNF-α cytokines contribute to the development of peripheral diabetic neuropathy (PDN) by promoting neuroinflammation, oxidative stress, and apoptosis of neurons [12]. PDN, the appearance of persistent wounds with the activation of the immune system, and the level of anti- and pro-inflammatory cytokines such as IL-6, TNF-α, and CRP considerably influence the wound-healing process [38,39]. Moreover, the increased level of these cytokines has already been reported to be associated with the occurrence of T2DM by modulating insulin signaling pathways favoring insulin resistance [25,40].

A study with a relatively small number of cases reported positive correlation between IL-6 levels, transaminase enzymes (ALT, AST), and GGT in patients with chronic hepatitis. The same study found no correlation between TNF-α levels and the measured biochemical parameters, opposite to our findings. This difference might be due to the specific pathology of the enrolled patients, elevated IL-6 being associated with a variety of liver diseases [41].

In this current study, TNF-α serum levels did not demonstrate statistical significance, which contrasts with the expected outcomes based on the most recent literature. In a previous study by Mauro et al., the upregulation of cytokines such as IL-6, TNF-α, and CRP was observed in patients undergoing major amputation, and a negative correlation with patient age was reported. To establish a clearer understanding of the role of TNF-α, a more homogeneous study group may be required [42].

The major goal of postoperative patient monitoring is prevention of complications. Intense postoperative inflammation can trigger arrhythmias, such as atrial fibrillation and ischemia–reperfusion injury can induce oxidative stress, which can worsen the outcome [43]. This study showed slightly elevated serum MDA levels after surgery compared to preoperative levels, and the positive correlation between lipid peroxidation and TNF-α levels suggests a close association between oxidative stress and inflammation. Several studies on metabolic and cardiovascular pathologies showed a similar relationship between these markers [44,45].

In a recent study, serum IL-6 was found to be the most effective predictor of disease severity in patients diagnosed with COVID-19 pneumonia. The authors considered critical levels of IL-6 over 35 pg/mL, and levels exceeding this threshold were suggestive of severe outcome and increased mortality [46]. Almost one quarter of the patients involved in the present study had critically elevated serum IL-6 levels based on their preoperative test results, which could influence the occurrence of perioperative complications. This statement is supported by the significant difference in the distribution of cases with prolonged hospitalization, hospital stays over 2 weeks being more frequent in the group of patients with critical preoperative IL-6 values. Based on our findings, this parameter can be considered a prognostic factor for complicated postoperative evolution in patients with vasculopathy. The proportion of patients with critically high IL-6 values was considerably lower two days after surgery, probably due to the administered medication.

The NLR did not turn out to be a useful prognostic marker for our patients; moreover, patients with normal values of this parameter had significantly longer hospitalization periods compared to those with values over 3. These results are in contradiction with other studies performed on similar patient populations [47]. A study performed in Italy on 324 elderly patients showed NLR over 2.4 to be a strong predictor of the presence of carotid plaques, better than routine inflammatory markers such as fibrinogen or CRP [48]. Taking into account the limited number of subjects, the interpretation of our findings should be made with caution. It is also important to mention that some patients (n = 3) had an extremely high NLR that exceeded 10, which had a major influence on the statistical findings. Preoperative IL-6 level seems to be a more reliable prognostic marker in our patients, so its measurement should be recommended despite its higher costs.

Limitations of the study are represented by the relatively small number of cases (due to the overlapping of the first phase of the survey, the enrollment of the patients, with the COVID-19 pandemic), and the short follow-up period. Although NLR was recognized to be both a diagnostic and prognostic biomarker of atherosclerotic plaques, variability of results in this proof-of-concept study did not allow us to confirm the data of the literature.

The originality and importance of our research stems from the study design, which involves a comprehensive pre- and postoperative follow-up of patients with vasculopathy, including the measurement of two inflammatory markers (IL-6 and TNF-α) and the evaluation of the lipid peroxidation level that are not routinely assessed. Only a limited number of studies have investigated the levels of inflammatory markers during the perioperative period. The findings of our study could have significant clinical implications by improving the identification of high-risk patients undergoing lower limb surgery, suggesting a possible predictive factor (critically high preoperative IL-6 value) for unfavorable disease course, thereby enabling healthcare professionals to implement appropriate measures to prevent complications.

## 4. Materials and Methods

A prospective study was performed during 2020–2023 at the Clinical County Hospital in Târgu-Mureș on diabetic and non-diabetic patients with vasculopathy (n = 31) admitted to the General Surgery and Vascular Surgery Units. The study was approved by the Ethics Committee of the hospital and that of the “George Emil Palade” University of Medicine Pharmacy, Science, and Technology of Târgu Mureş. The patients signed an informed consent document regarding their participation in the study.

The inclusion criteria were the following: adult patients with vasculopathy requiring surgery of the lower limb admitted to these departments during the study period, willing to participate. Exclusion criteria: pregnancy, traumatic cause of morbidity. Demographic and clinical data were collected, including the length of hospitalization. Blood samples were collected before surgery (lower limb amputation or necrectomy) and 48 h after surgery (62 samples).

Routine hematological (complete blood count—CBC) and biochemical tests (glycemia, uricemia, lipid profile, transaminase and GGT activity, kidney function tests) were performed at the laboratory of the Clinical County Hospital on a Cell Dyn Ruby (Abbott Laboratories, Chicago, IL, USA) hematology analyzer and on an Architect c4000 (Abbott Laboratories, Chicago, IL, USA) biochemistry analyzer, besides some routine inflammatory markers. These routine inflammatory markers included: C-reactive protein measured by turbidimetric method on the same Architect c4000 equipment, erythrocyte sedimentation rate (ESR) measured by Westergren method and fibrinogen (Sysmex CS 2500 equipment, Sysmex Corporation, Kobe, Hyogo, Japan). Blood samples were collected in serum-separator tubes (SST) for routine biochemistry tests and on EDTA for CBC. Neurophil to Lymphocyte Ratio (NLR) was calculated for each of the patients based on their preoperative CBC results. Supplementary, after centrifugation, serum samples were frozen at −70 °C and preserved at this temperature at the Center for Advanced Medical and Pharmaceutical Research (CAMPhR) for inflammatory cytokine assessment and oxidative stress measurement. Assessment of TNF-α and IL-6 was performed on a Flexmap 3D (Luminex Corporation, Austin, TX, USA) analyzer using the xMAP technology at the CAMPhR, using the multiplex assay kit Milliplex Human Cytokine/Chemokine Magnetic Bead Panel (Millipore Sigma, Burlington, MA, USA). The evaluation of oxidative stress consisted of the assessment of serum malondialdehyde by a newly developed TBARS (thiobarbituric acid reactive substances) method using HPLC UV-VIS Thermo Surveyor equipment (Thermo Fischer Scientific Inc, Waltham, MA, USA), Supelcosil LC-18 columns (Sigma Aldrich, Chemie HmbH, Munich, Germany), with phosphate buffer as mobile phase (Merck KGaA, Darmstadt, Germany) [49].

SPSS version 28 and GraphPad InStat version 3 were used for the statistical processing of the obtained data. Paired and unpaired Student *t* test with and without Welch correction, Pearson’s correlation test, and chi-square test were used. The threshold of significance was set at *p* < 0.05. The Kolmogorov–Smirnov normality test was used for the evaluation of the Gaussian distribution of the data.

## 5. Conclusions

The dynamic assessment of inflammatory markers is appropriate for monitoring the outcome of patients undergoing surgery. Preoperative IL-6 concentrations were critically high in one quarter of the study group and moderately elevated in half of the subjects. There was a statistically significant difference in IL-6 levels at the postoperative follow-up in the diabetic subgroup compared with the non-diabetic subgroup. In this study, the predictive value of preoperative IL-6 serum levels was observed, as subjects with IL-6 levels above 35 pg/mL required hospitalization for more than 2 weeks. The measurement of several inflammatory markers (IL-6, PCR) and biochemical parameters can be useful in the identification of patients predisposed to complications, which can benefit from more complex management.

## Figures and Tables

**Table 1:** Preoperative values followed by postoperative values.

Parameter	SGL	STC	HDL	STG	ALT	AST	GGT	SUA	SUR	SCR
Preoperative values										
Unit	mg/dL	mg/dL	mg/dL	mg/dL	IU/L	IU/L	IU/L	mg/dL	mg/dL	mg/dL
Mean	147.34	161.13	34.85	144.18	26.96	21.86	74.52	6.39	51.31	1.32
±SD	94.87	52.69	10.54	80.65	45.78	19.36	134.56	2.19	35.15	1.69
Postoperative values										
Unit	mg/dL	mg/dL	mg/dL	mg/dL	IU/L	IU/L	IU/L	mg/dL	mg/dL	mg/dL
Mean	131.13	153.57	35.43	152.75	64.84	47.22	74.85	6.16	47.54	1.27
±SD	66.87	45.30	8.14	78.53	199.02	84.40	140.71	2.42	37.57	1.77
Significance of difference between pre- and postoperative values										
*p*-value	0.4428	0.5593	0.8362	0.6918	0.3100	0.1188	0.9926	0.7124	0.6873	0.9111

SGL—serum glucose level; STC—serum total cholesterol; HDL—high density lipoproteins; STG—serum triglycerides; ALT—alanine amino transferase; AST—aspartate amino transferase; GGT—gamma glutamyl transpeptidase; SUA—serum uric acid; SUR—serum urea; SCR—serum creatinine.

**Table 2:** Relationship between postoperative liver function tests and TNF-α in patients with vasculopathy.

Parameters	Postoperative ALT	Postoperative AST	Postoperative GGT
Postoperative TNF-α	r = 0.621 *p* < 0.001	r = 0.513 *p* = 0.004	r = 0.678 *p* < 0.001

ALT—alanine amino transferase; AST—aspartate amino transferase; GGT—gamma glutamyl transpeptidase.

## Data Availability

Data is contained within the article.
